# Exome chip association study excluded the involvement of rare coding variants with large effect sizes in the etiology of anorectal malformations

**DOI:** 10.1371/journal.pone.0217477

**Published:** 2019-05-28

**Authors:** Romy van de Putte, Charlotte H. W. Wijers, Heiko Reutter, Sita H. Vermeulen, Carlo L. M. Marcelis, Erwin Brosens, Paul M. A. Broens, Markus Homberg, Michael Ludwig, Ekkehart Jenetzky, Nadine Zwink, Cornelius E. J. Sloots, Annelies de Klein, Alice S. Brooks, Robert M. W. Hofstra, Sophie A. C. Holsink, Loes F. M. van der Zanden, Tessel E. Galesloot, Paul Kwong-Hang Tam, Marloes Steehouwer, Rocio Acuna-Hidalgo, Maartje van de Vorst, Lambertus A. Kiemeney, Maria-Mercè Garcia-Barceló, Ivo de Blaauw, Han G. Brunner, Nel Roeleveld, Iris A. L. M. van Rooij

**Affiliations:** 1 Department for Health Evidence, Radboud Institute for Health Sciences, Radboud University Medical Center, Nijmegen, The Netherlands; 2 Institute of Human Genetics, University of Bonn, Bonn, Germany; 3 Department of Neonatology, Children’s Hospital, University of Bonn, Bonn, Germany; 4 Department of Human Genetics, Radboud Institute for Molecular Life Sciences, Radboud University Medical Center, Nijmegen, The Netherlands; 5 Department of Clinical Genetics, Erasmus Medical Centre, Rotterdam, The Netherlands; 6 Department of Pediatric Surgery, Sophia’s Children’s Hospital—Erasmus Medical Centre, Rotterdam, The Netherlands; 7 Department of Surgery, Division of Pediatric Surgery, University Medical Center Groningen, Groningen, The Netherlands; 8 Department of Child and Adolescent Psychiatry and Psychotherapy, Johannes-Gutenberg University, Mainz, Germany; 9 Department of Clinical Chemistry and Clinical Pharmacology, University of Bonn, Bonn, Germany; 10 Division of Clinical Epidemiology and Aging Research, German Cancer Research Center, Heidelberg, Germany; 11 Department of Surgery, Li Ka Shing Faculty of Medicine of the University of Hong Kong, Hong Kong, China; 12 Centre for Reproduction, Development and Growth, Li Ka Shing Faculty of Medicine of the University of Hong Kong, Hong Kong, China; 13 Department of Psychiatry, Li Ka Shing Faculty of Medicine of the University of Hong Kong, Hong Kong, China; 14 Department of Urology, Radboud Institute for Health Sciences, Radboud University Medical Center, Nijmegen, The Netherlands; 15 Experimental Cardiology Laboratory, Division Heart and Lungs, University Medical Center Utrecht, Utrecht, the Netherlands; 16 Image Sciences Institute, University Medical Center Utrecht, Utrecht, The Netherlands; 17 Department of Surgery—Pediatric Surgery, Radboudumc Amalia Children’s Hospital, Radboud University Medical Center, Nijmegen, The Netherlands; 18 Department of Clinical Genetics, Maastricht University Medical Centre, Maastricht, The Netherlands; Oslo Universitetssykehus, NORWAY

## Abstract

**Introduction:**

Anorectal malformations (ARM) are rare congenital malformations, resulting from disturbed hindgut development. A genetic etiology has been suggested, but evidence for the involvement of specific genes is scarce. We evaluated the contribution of rare and low-frequency coding variants in ARM etiology, assuming a multifactorial model.

**Methods:**

We analyzed 568 Caucasian ARM patients and 1,860 population-based controls using the Illumina HumanExome Beadchip array, which contains >240,000 rare and low-frequency coding variants. GenomeStudio clustering and calling was followed by re-calling of ‘no-calls’ using zCall for patients and controls simultaneously. Single variant and gene-based analyses were performed to identify statistically significant associations, applying Bonferroni correction. Following an extra quality control step, candidate variants were selected for validation using Sanger sequencing.

**Results:**

When we applied a MAF of ≥1.0%, no variants or genes showed statistically significant associations with ARM. Using a MAF cut-off at 0.4%, 13 variants initially reached statistical significance, but had to be discarded upon further inspection: ten variants represented calling errors of the software, while the minor alleles of the remaining three variants were not confirmed by Sanger sequencing.

**Conclusion:**

Our results show that rare and low-frequency coding variants with large effect sizes, present on the exome chip do not contribute to ARM etiology.

## Introduction

Congenital anorectal malformations (ARM) are the most frequent malformations of the gastrointestinal tract, with a prevalence of 2 to 7 in 10,000 live births[1]. ARM encompass a broad range of phenotypes, which are usually classified according to the type of fistula to neighboring organs. In approximately 50% of the patients, ARM is associated with additional congenital malformations, including vertebral, cardiac and/or renal malformations[2, 3]. Multiple surgical corrections are required during the first years of a patient’s life. Despite major improvements in treatment and care of ARM patients in the past decade, a substantial number of patients face lifelong physical and psychosocial problems[4].

Our current understanding of the embryology and etiology of ARM is limited. Syndromes caused by a fully penetrant mutation in a single gene, such as Currarino syndrome (OMIM #176450) or Townes-Brocks syndrome (OMIM #107480), are identified in at most 10% of ARM patients[2]. In the remaining patients, the involvement of both genetic and non-genetic factors in the occurrence of ARM seems likely. Aggregation of ARM has been observed in some families[5–8]. In addition, non-genetic risk factors, such as fertility treatment[9–12], maternal overweight or obesity[5, 13, 14], pre-existing diabetes[15–17], and previous miscarriages[18], have all been found to be associated with ARM in various studies. Genetic research into ARM has mainly focused on candidate genes that are involved in embryonic signaling pathways, such as sonic hedgehog (*SHH*), wingless-type integration site (*WNT*), and fibroblast growth factor (*FGF*) signaling, identified from animal studies[19, 20]. Human ARM studies provided only limited evidence to support a contribution of these genes in ARM etiology[21–28]. Hypothesis-generating approaches, such as genome-wide studies seem valuable to obtain new knowledge and hypotheses on genes being involved in the etiology of ARM.

Until now, Wong *et al*. performed the only genome-wide association study for ARM in 175 ARM patients. This study did not yield any associated common single nucleotide variants, and the results did not suggest a role for common copy number variants (CNV) in the etiology of ARM either[29]. There was, however, an apparent excess of rare CNVs in ARM patients compared to controls and to healthy individuals from the Database of Genomic Variants[29]. This suggests the importance of rare genomic variation in the etiology of ARM.

In this study, we aimed to identify rare genetic variants for ARM by genotyping >240,000 known rare and low-frequency coding variants, using an Illumina Human Exome Beadchip array, in a cohort of almost 600 ARM patients.

## Materials and methods

### Study population

AGORA (Aetiologic research into Genetic and Occupational/environmental Risk factors for Anomalies in children) is a large data- and biobank with DNA samples and clinical and questionnaire data from children with congenital malformations or childhood cancer, control children, and their parents. AGORA is a multicentre effort coordinated by the Radboud university medical center (Radboudumc) in Nijmegen, The Netherlands[30]. For the current study, AGORA provided 429 blood or saliva samples from live born European patients who were treated for ARM at the departments of Pediatric Surgery of the Radboudumc Amalia Children’s Hospital, the Sophia Children’s Hospital Erasmus MC Rotterdam (EMC), and the University Medical Center Groningen (UMCG), in The Netherlands. The German Network for Congenital Uro-REctal malformations (CURE-Net) provided 169 additional DNA samples from ARM patients of European ancestry. These patients were recruited through the German self-help organization for ARM patients (SoMA e.V.) and pediatric surgical departments throughout Germany. DNA samples from ARM patients with known chromosomal abnormalities or syndromes with a known genetic cause were excluded from the study population.

Pediatric surgeons, clinical geneticists, and researchers reviewed the medical records of the ARM patients extensively to obtain clinical information on ARM phenotypes and associated birth defects. We classified ARM phenotypes according to the Krickenbeck criteria[31], while other congenital malformations were classified according to the EUROCAT classification[32].

Controls were derived from the Nijmegen Biomedical Study (NBS), a population-based survey conducted by the Department for Health Evidence and the Department of Laboratory Medicine of the Radboudumc[33]. In total, 22,451 age and sex stratified randomly selected adult inhabitants of the municipality of Nijmegen received an invitation to fill out a postal questionnaire on items, such as lifestyle and medical history, and to donate two blood samples. The response to the questionnaire was 43% (N = 9,350), and 69% (N = 6,468) of the responders donated blood samples. For the current study, DNA samples of 1,930 European controls were used. The Arnhem-Nijmegen Regional Committee on Research Involving Human Subjects approved the AGORA and NBS study protocols and the Ethics Committees of the University of Bonn and the University of Heidelberg approved the CURE-Net study protocol. Written informed consent was obtained from all adult participants and parental consent for children under 18 years of age.

### Genotyping and quality control

Using standard methods, DNA was extracted from blood collected in EDTA-containing tubes or saliva specimens collected in Oragene containers (DNA Genotek Inc., Ottawa, Canada). The DNA samples were genotyped using the Illumina HumanExome BeadChip array (v1.1), which contains 242,901 variants throughout the exome, hereafter referred to as exome chip. The majority of these variants (~220,000) are rare and low-frequency coding variants (nonsynonymous, splice site, stop gain, and stop loss variants), and a small proportion are non-coding variants. The variants were selected based on their occurrence in approximately 12,000 sequenced genomes and exomes from multiple populations of primarily European ancestry[34]. Approximately 20% of the variants have a minor allele frequency (MAF) >1%, and 80% of the variants a MAF <1%. Genotyping, calling, and quality control procedures were performed at the Erasmus MC in Rotterdam, The Netherlands, as part of the ExomeChip Rainbow Project (RP10) of Biobanking and Biomolecular Research Infrastructure Netherlands (BBMRI-NL). Regular GenomeStudio clustering and calling was followed by quality control and re-calling of the ‘no-calls’ using zCall, for patient and control samples simultaneously, to prevent batch effects. The zCall tool was especially designed for calling rare variants from array data[35].

The raw dataset after GenomeStudio calling consisted of 2,528 samples and 242,532 variants. After quality control (S1 Fig), 2,432 samples and 239,253 variants remained before the zCall procedure was applied. After the zCall procedure, two additional samples were removed because of excess heterozygosity, 205 additional variants because of low call rates (<99%), and 6 other variants because they were not in Hardy-Weinberg equilibrium (HWE). In total, 2,430 samples and 239,042 variants were included in the final dataset. We additionally removed one patient because of a clinical diagnosis of caudal regression syndrome (OMIM #600145) and one patient because of known relatedness with another patient (PI-HAT 0.28). As a result, 239,042 genotyped variants in 568 patients and 1,860 controls remained for further analyses.

### Statistical analyses

Statistical analyses were performed using PLINK v1.07, RStudio v.0.99.903, and SPSS v22.0.

#### Single variant analyses

We first restricted the single variant analyses to variants with a minor allele frequency (MAF) value of ≥1.0% in the combined patient and control series. Based on this criterion, 36,062 markers remained for the statistical analyses. Secondly, we were interested in less frequently occurring variants. To reduce potential false-positives and to have sufficient power to possibly replicate a statistically significantly associated variant we investigated variants with a MAF value of ≥0.4%, an arbitrary value which corresponds to a minor allele count of a minimum of 20 minor alleles in the combined patient and control series. Based on this criterion, 43,653 markers remained for the statistical analyses.

We tested the associations between ARM and each variant using Fisher’s exact tests in an allelic model, assuming HWE, and an additive genetic model. We used an exact method, as the asymptotic score test is known to be too liberal and does not provide robust results when variant allele counts are low. Potential of bias due to population stratification was assessed using the quantile-quantile (QQ) plot and by calculating the genomic inflation factor (lambda), which was defined as the regression coefficient of the observed to expected–log *p* values from the Fisher’s exact test. Test statistics were adjusted for lambda. A *p*-value below 1.39x10^-6^ was considered statistically significant in the single variant analyses with a MAF value of ≥1.0%, which corresponds to a *p*-value of 0.05 with Bonferroni correction for 36,062 tests. Similarly, a *p*-value below 1.15x10^-6^ with Bonferroni correction for 43,653 tests was used in the single variant analyses with a MAF ≥0.4%.

#### Gene-based analyses

Gene-based analyses were performed using the sequence kernel association test (SKAT) in R. SKAT has been shown to be a powerful method when both harmful and protective variants with different magnitudes of effect occur in one gene[36]. We tested 6,188 genes with at least two variants that passed quality control and were polymorphic in either patients or controls. A *p-*value below 8.08x10^-6^ was considered statistically significant, corresponding to a Bonferroni correction for 6,188 tests. The analyses were performed using default settings[36].

### Extra quality control step

To make sure that the statistically significant variants represented true positive calls, we applied an extra quality control step (S1 Text) for the variants that showed apparent statistical significance in the single variant analyses. This involved manual inspection of the minor allele calls in the cluster plots to exclude possible technical artefacts, which may have led to false calls.

### Sanger sequencing

After the extra quality control step, three SNPs were selected for technical validation using Sanger Sequencing, including all samples that showed a heterozygous genotype for one of these three SNPs. More detailed information on primer design and Sanger sequencing can be found in S1 Text. The sequences obtained were compared with the reference sequence derived from the UCSC genome browser (Hg38) by two independent researchers using VECTOR NTI software 11.0.

## Results

### ARM patients and controls

In total, exome chip data on 568 ARM patients and 1,860 controls passed quality control criteria and were included in the analyses. The majority of patients had a perineal fistula (42%), followed by rectourethral fistulas (20%), vestibular fistulas (16%), cloaca (6%), ARM without fistula (5%), rectovesical fistula (3%), anal stenosis (2%), and rare/other types of ARM (2%). Isolated ARM was observed among 306 patients (54%), while 257 patients (46%) had one or more additional major congenital malformation (Table 1).

**Table 1:** Gender of patients and controls and detailed information on the phenotype of the patients (type of anorectal malformation and presence of additional congenital malformations).

	ARM patients^a^ (n = 568) No. (%)	Controls^a^ (n = 1,860) No. (%)
**Gender**		
Male	299 (52.6)	918 (49.4)
Female	269 (47.4)	942 (50.6)
**Phenotypes of ARM**		
Perineal fistula	238 (41.9)	
Vestibular fistula	92 (16.2)	
ARM without fistula	30 (5.3)	
Rectourethral fistula		
*Bulbar*	44 (7.7)	
*Prostate*	38 (6.7)	
*Unspecified*	34 (6.0)	
Rectovesical fistula	18 (3.2)	
Cloaca	32 (5.6)	
Anal stenosis	12 (2.1)	
Rare types^b^	13 (2.3)	
Type unknown	17 (3.0)	
**Subgroups of ARM**		
Isolated ARM	306 (53.9)	
ARM with other congenital malformations	257 (45.2)	
Unknown	5 (0.9)	

ARM: anorectal malformations.

^a^ All patients and controls were European.

^b^ Including rectovaginal fistula, rectal atresia, rectal stenosis, and dorsal cloaca-like defect with complex H-fistula.

### Statistical analyses—Single variant analyses

Single variant analyses using an allelic model (lambda 1.063) for variants with MAF ≥1% did not identify variants that were statistically significantly associated with ARM (Figure A in S2 Fig).

Analyses of variants with MAF ≥0.4% and an allelic model identified 13 variants that were statistically significantly associated with ARM (lambda 1.068) (Table 2 and Figure B in S2 Fig). The additive genotype model was also applied, and showed similar results, after correction of lambda 1.072 (S1 Table).

**Table 2:** Statistically significant (but false positive) associations between anorectal malformations and single variants (MAF value ≥ 0.4%).

ID	Chr	Position^a^	rs-id	Minor / Major allele	Protein change	Gene	MAF Controls (N = 1,860) %	MAF patients (N = 568) %	*P*^b^	Population Frequency^c^ (%) (allele number)
exm1082598	13	114537621	rs78824256	T/C	Arg246His	*GAS6*^d^, *GAS6-AS1*	0	2.664	1.18x10^-18^	0.0064 (62522)
exm42669	1	34330070	rs144223004	T/C	Pro93Leu	*HMGB4*^d^, *CSMD2*	0	2.553	5.71x10^-18^	0.0000 (66366)
exm1576973	21	45821582	rs9974927	G/T	Asp780Glu	*TRPM2*	0.054	2.748	6.85x10^-17^	0.1023 (65498)
exm2117113	6	169642042	rs202062355	T/C	Ala236Thr	*THBS2*	0	2.381	8.67x10^-17^	0.0016 (63516)
exm876692	11	1281885	rs113740363	G/A	Ile5666Val	*MUC5B*	0	2.377	8.98x10^-17^	0.0016 (62600)
exm870455	11	403981	rs202227463	A/G	Gly721Ser	*PKP3*	0.027	2.504	3.03x10^-16^	0.0031 (64534)
exm1093644	14	24780216	rs201778907	G/A	Ser116Gly	*CIDEB*, *LTB4R2*^d^	0.027	2.465	4.19x10^-16^	0.0000 (55448)
exm297681	3	33195264	rs75287757	T/C	Arg287Gln	*SUSD5*	0	2.201	1.41x10^-15^	0.0150 (66628)
exm1452159	19	32844890	rs150068736	G/T	Ile385Ser	*ZNF507*	0	1.849	3.44x10^-13^	0.0271 (66542)
exm603	1	900519	rs140019196	G/A	Asn626Ser	*KLHL17*	0	1.761	1.36x10^-12^	0.0000 (65016)
exm1510860	19	56487619	rs61734100	G/C	Ile942Met	*NLRP8*	0.108	2.025	8.16x10^-11^	0.1125 (66654)
exm346857	3	126236523	rs147828466	A/G	Arg14Trp	*UROC1*	0.242	2.347	6.31x10^-10^	0.2218 (31112)
exm1560265	20	62324609	rs139221232	T/C	Arg989Trp	*RTEL1*	0.188	1.645	5.65x10^-7^	0.0292 (65178)

Chr: chromosome; MAF: minor allele frequency;

^a^ Genome positions are based on human genome build hg19;

^b^ *P* value calculated for an **allelic model** using Fisher’s exact test and adjusted for genomic control (lambda = 1.068);

^c^ The population frequency (%) in the European (Non-Finnish) population, according to the Exome Aggregation Consortium (29);

^d^ Variant affects the coding region of this gene, but intronic region in other gene.

### Extra quality control step

The extra quality control step for the 13 statistically significant variants from the single variant analysis (MAF ≥0.4%) showed that 10 out of the 13 variants most likely represented false minor allele calls due to technical errors (S1 Text). Therefore, these variants were excluded as candidates for technical validation using Sanger sequencing.

### Sanger sequencing

The three variants that remained eligible for technical validation using Sanger sequencing were exm42669, exm297681, and exm1452159. For variant exm42669, we were unable to confirm heterozygosity in 28 out of the 29 patients who were called as heterozygous risk allele carriers by the exome chip. We did not have DNA available for the last patient anymore. For variant exm297681, we had DNA for 22 out of 24 patients available and for variant exm1452159, for 29 out of 31 patients. Again, we were not able to confirm the presence of the minor alleles for these variants by Sanger sequencing.

### Statistical analyses—Gene-based analyses

Gene-based analyses were performed for the dataset with MAF restricted to ≥1.0%, as we encountered many false-positive findings when we restricted the MAF cut-off to ≥0.4%. We did not find any gene that was statistically significantly associated with ARM in the combined patient and control series. The genes that passed a *p-*value threshold of 0.01 are given in S2 Table.

## Discussion

This study aimed to identify rare genetic variants associated with ARM by analyzing exome chip data of >240,000 known rare and low-frequency coding variants in a large ethnically homogeneous series of 568 Caucasian ARM patients and 1,860 Caucasian population-based controls. We identified 13 variants that were statistically significantly associated with ARM, but all of these appeared to be false-positive findings. Therefore, this first exome chip study did not provide statistical evidence for association of rare genetic variants with large effect sizes and ARM.

Although the study by Wong *et al*. suggested the importance of rare genomic variation in the etiology of ARM[29], we were not able to support this hypothesis with findings in the current study. Although the prevalence of ARM is relatively low, we were able to include a large number of patients. The frequency of ARM patients with additional anomalies in our study was in concordance with previous literature[2, 3, 11], and the distribution of ARM phenotypes was comparable with patients from the European ARM-Net registry[37]. This illustrates that we included a representative ARM patient series within our study. Despite the inclusion of almost 600 ARM patients, we could only detect, at 80% power and a multiple testing-adjusted alpha, rare variants with MAFs of 0.4–1.0% that have large effect on ARM (allelic ORs >4.0). To be able to detect rare variants with smaller effect sizes (e.g. OR 2.0), >3000 ARM patients are required for variants with MAF 1.0% and >7500 ARM patients for variants with MAF 0.4%. Because of the assumption of a multifactorial model in ARM etiology, smaller effect sizes are likely, which indicates the need for much larger sample sizes when arrays are used.

Given the well-known limitations of calling rare variants on genotyping arrays[38], the higher risk of incorrect genotype calling when lower MAFs are used[39], and the large number of rare variants that were present on the exome chip, measurement errors may have had a large impact on the study results. However, we applied Bonferroni corrections to minimize the chance of reporting false-positive associations. In addition, we applied extra quality control steps, which included the inspection of the cluster plots and validation by Sanger sequencing. Both of these quality control steps suggested that the initial findings for all 13 statistically significant variants were driven by incorrect calling of rare alleles. This highlights the importance of visual inspection of cluster plots and validation of findings using an independent genotyping technique.

Another note about this study is that the content of the exome chip is based on the coding and splice site variants that were observed several times in approximately 12,000 sequenced individuals of mainly European descent. Hence, a large proportion of rare human exome variation and private variant alleles were not covered. Therefore, whole genome sequencing is required to allow for discovering relevant rare coding variants that are not covered by this exome chip, as well as relevant non-coding variants. Because ARM is observed as a component within certain syndromes[2], and aggregation of ARM has been observed within families[5–8], a role of genetics in the etiology of ARM remains likely.

Wong *et al*. performed the only GWAS thus far in 175 cases and 2971 controls, which failed to detect common variants associated with ARM[29]. Both, the study by Wong *et al*. and our study indicate that major effects of rare or common single nucleotide variants as captured by exome- and genome-wide arrays do not seem to play a major role in the etiology of ARM. ARM appears to comprise a genetically heterogeneous set of malformations, as was also suggested by Wong *et al*.[40] A potential hypothesis regarding the etiology is that ARM may only be caused by single-gene defects in a, probably small, proportion of patients. Since ARM comprise a wide spectrum of phenotypes, several different monogenic forms of ARM may exist. This hypothesis was also suggested for congenital malformations of the kidney and urinary tract (CAKUT)[41]. Equally, ARM may be a truly multifactorial disorder with involvement of many genetic as well as non-genetic risk factors with relatively small effects acting simultaneously in the majority of patients. Support for this scenario derives from several non-genetic factors being associated with ARM[19],[42].

In conclusion, the present study among 568 ARM patients did not yield evidence for associations between ARM and rare and low-frequency coding variants with large effect sizes, as captured by the exome chip. Future studies with even larger sample sizes are needed to identify potential common and/or rare genetic variants with small effects on ARM etiology. For these studies, international collaborations are essential. Genetic studies in phenotypically homogenous subgroups of ARM may further contribute to the elucidation of underlying genetic causes.

## Supporting information

S1 Fig Summary of the quality control steps before the zCall procedure was applied. (PDF)

S2 Fig Manhattan plots. (PDF)

S3 Fig Cluster plots. (PDF)

S1 Table Statistically significant (but false positive) associations between anorectal malformations and single variants (MAF value ≥ 0.4%), genotypic model. (DOCX)

S2 Table Associations between anorectal malformations and genes with at least two variants with a MAF ≥ 1.0%. (DOCX)

S1 Text Additional information on methods and results. (DOCX)

## References

[1] InternationalClearinghouse. Annual report 2014—International clearinghouse for birth defects surveillance and research. 2014.

[2] Cuschieri A. Anorectal anomalies associated with or as part of other anomalies. Am J Med Genet. 2002;110(2):122–30. Epub 2002/07/13. 10.1002/ajmg.10371 . 12116249

[3] Stoll C, Alembik Y, Dott B, Roth MP. Associated malformations in patients with anorectal anomalies. Eur J Med Genet. 2007;50(4):281–90. Epub 2007/06/19. 10.1016/j.ejmg.2007.04.002 . 17572165

[4] Hartman EE, Oort FJ, Aronson DC, Hanneman MJ, van der Zee DC, Rieu PN, et al Critical factors affecting quality of life of adult patients with anorectal malformations or Hirschsprung’s disease. Am J Gastroenterol. 2004;99(5):907–13. Epub 2004/05/07. 10.1111/j.1572-0241.2004.04149.x . 15128359

[5] van Rooij IA, Wijers CH, Rieu PN, Hendriks HS, Brouwers MM, Knoers NV, et al Maternal and paternal risk factors for anorectal malformations: a Dutch case-control study. Birth Defects Res A Clin Mol Teratol. 2010;88(3):152–8. Epub 2010/01/15. 10.1002/bdra.20649 . 20073076

[6] Falcone RA Jr., Levitt MA, Pena A, Bates M. Increased heritability of certain types of anorectal malformations. J Pediatr Surg. 2007;42(1):124–7; discussion 7–8. Epub 2007/01/09. 10.1016/j.jpedsurg.2006.09.012 . 17208552

[7] Dworschak GC, Zwink N, Schmiedeke E, Mortazawi K, Marzheuser S, Reinshagen K, et al Epidemiologic analysis of families with isolated anorectal malformations suggests high prevalence of autosomal dominant inheritance. Orphanet J Rare Dis. 2017;12(1):180 10.1186/s13023-017-0729-7 . 29237507 PMC5729416

[8] Teerlink CC, Bernhisel R, Cannon-Albright LA, Rollins MD. A genealogical assessment of familial clustering of anorectal malformations. J Hum Genet. 2018;63(10):1029–34. Epub 2018/07/08. 10.1038/s10038-018-0487-y . 29980720

[9] Reefhuis J, Honein MA, Schieve LA, Correa A, Hobbs CA, Rasmussen SA. Assisted reproductive technology and major structural birth defects in the United States. Hum Reprod. 2009;24(2):360–6. Epub 2008/11/18. 10.1093/humrep/den387 . 19010807

[10] Zwink N, Jenetzky E, Schmiedeke E, Schmidt D, Marzheuser S, Grasshoff-Derr S, et al Assisted reproductive techniques and the risk of anorectal malformations: a German case-control study. Orphanet J Rare Dis. 2012;7:65 10.1186/1750-1172-7-65 . 22978793 PMC3519554

[11] Wijers CH, van Rooij IA, Bakker MK, Marcelis CL, Addor MC, Barisic I, et al Anorectal malformations and pregnancy-related disorders: a registry-based case-control study in 17 European regions. BJOG. 2013;120(9):1066–74. Epub 2013/04/12. 10.1111/1471-0528.12235 . 23574029

[12] Wijers CH, van Rooij IA, Rassouli R, Wijnen MH, Broens PM, Sloots CE, et al Parental subfertility, fertility treatment, and the risk of congenital anorectal malformations. Epidemiology. 2015;26(2):169–76. Epub 2015/01/08. 10.1097/EDE.0000000000000226 . 25563433

[13] Blomberg MI, Kallen B. Maternal obesity and morbid obesity: the risk for birth defects in the offspring. Birth Defects Res A Clin Mol Teratol. 2010;88(1):35–40. Epub 2009/08/28. 10.1002/bdra.20620 . 19711433

[14] Waller DK, Shaw GM, Rasmussen SA, Hobbs CA, Canfield MA, Siega-Riz AM, et al Prepregnancy obesity as a risk factor for structural birth defects. Arch Pediatr Adolesc Med. 2007;161(8):745–50. Epub 2007/08/08. 10.1001/archpedi.161.8.745 . 17679655

[15] Correa A, Botto L, Liu Y, Mulinare J, Erickson JD. Do multivitamin supplements attenuate the risk for diabetes-associated birth defects? Pediatrics. 2003;111(5 Pt 2):1146–51. Epub 2003/05/03. . 12728128

[16] Correa A, Gilboa SM, Besser LM, Botto LD, Moore CA, Hobbs CA, et al Diabetes mellitus and birth defects. Am J Obstet Gynecol. 2008;199(3):237 e1-9. 10.1016/j.ajog.2008.06.028 . 18674752 PMC4916956

[17] Frias JL, Frias JP, Frias PA, Martinez-Frias ML. Infrequently studied congenital anomalies as clues to the diagnosis of maternal diabetes mellitus. Am J Med Genet A. 2007;143A(24):2904–9. Epub 2007/11/15. 10.1002/ajmg.a.32071 . 18000913

[18] van de Putte R, Wijers CH, de Blaauw I, Marcelis CL, Sloots CE, Brooks AS, et al Previous miscarriages and GLI2 are associated with anorectal malformations in offspring. Hum Reprod. 2017;32(2):299–306. Epub 2017/01/07. 10.1093/humrep/dew327 . 28057877

[19] Wijers CH, van Rooij IA, Marcelis CL, Brunner HG, de Blaauw I, Roeleveld N. Genetic and nongenetic etiology of nonsyndromic anorectal malformations: a systematic review. Birth Defects Res C Embryo Today. 2014;102(4):382–400. Epub 2014/12/30. 10.1002/bdrc.21068 . 25546370

[20] Khanna Kashish, Sharma Shilpa, Pabalan Noel, Singh Neetu, Gupta DK. A review of genetic factors contributing to the etiopathogenesis of anorectal malformations. Pediatric Surgery International. 2017:1–12. 10.1007/s00383-017-4204-2 29094201

[21] Seri M, Martucciello G, Paleari L, Bolino A, Priolo M, Salemi G, et al Exclusion of the Sonic Hedgehog gene as responsible for Currarino syndrome and anorectal malformations with sacral hypodevelopment. Hum Genet. 1999;104(1):108–10. Epub 1999/03/10. . 10071202 10.1007/s004390050919

[22] Garcia-Barcelo MM, Chi-Hang Lui V, Miao X, So MT, Yuk-yu Leon T, Yuan ZW, et al Mutational analysis of SHH and GLI3 in anorectal malformations. Birth Defects Res A Clin Mol Teratol. 2008;82(9):644–8. Epub 2008/07/26. 10.1002/bdra.20482 . 18655123

[23] Carter TC, Kay DM, Browne ML, Liu A, Romitti PA, Kuehn D, et al Anorectal atresia and variants at predicted regulatory sites in candidate genes. Ann Hum Genet. 2013;77(1):31–46. 10.1111/j.1469-1809.2012.00734.x . 23127126 PMC3535506

[24] Papapetrou C, Drummond F, Reardon W, Winter R, Spitz L, Edwards YH. A genetic study of the human T gene and its exclusion as a major candidate gene for sacral agenesis with anorectal atresia. J Med Genet. 1999;36(3):208–13. . 10204846 PMC1734318

[25] Kruger V, Khoshvaghti M, Reutter H, Vogt H, Boemers TM, Ludwig M. Investigation of FGF10 as a candidate gene in patients with anorectal malformations and exstrophy of the cloaca. Pediatr Surg Int. 2008;24(8):893–7. Epub 2008/07/01. 10.1007/s00383-008-2193-x . 18587586

[26] Draaken M, Prins W, Zeidler C, Hilger A, Mughal SS, Latus J, et al Involvement of the WNT and FGF signaling pathways in non-isolated anorectal malformations: sequencing analysis of WNT3A, WNT5A, WNT11, DACT1, FGF10, FGFR2 and the T gene. Int J Mol Med. 2012;30(6):1459–64. Epub 2012/09/11. 10.3892/ijmm.2012.1124 . 22961180

[27] van de Putte R, Wijers CH, de Blaauw I, Feitz WF, Marcelis CL, Hakobjan M, et al Sequencing of the DKK1 gene in patients with anorectal malformations and hypospadias. Eur J Pediatr. 2015;174(5):583–7. Epub 2014/10/17. 10.1007/s00431-014-2436-x . 25319845

[28] Zhang J, Zhang ZB, Gao H, Zhang D, Wang WL. Down-regulation of SHH/BMP4 signalling in human anorectal malformations. J Int Med Res. 2009;37(6):1842–50. Epub 2010/02/12. 10.1177/147323000903700620 . 20146882

[29] Wong EH, Cui L, Ng CL, Tang CS, Liu XL, So MT, et al Genome-wide copy number variation study in anorectal malformations. Hum Mol Genet. 2013;22(3):621–31. Epub 2012/10/31. 10.1093/hmg/dds451 . 23108157

[30] van Rooij IA, van der Zanden LF, Bongers EM, Renkema KY, Wijers CH, Thonissen M, et al AGORA, a data- and biobank for birth defects and childhood cancer. Birth Defects Res A Clin Mol Teratol. 2016;106(8):675–84. Epub 2016/05/07. 10.1002/bdra.23512 . 27150573

[31] Holschneider A, Hutson J, Pena A, Beket E, Chatterjee S, Coran A, et al Preliminary report on the International Conference for the Development of Standards for the Treatment of Anorectal Malformations. J Pediatr Surg. 2005;40(10):1521–6. Epub 2005/10/18. 10.1016/j.jpedsurg.2005.08.002 . 16226976

[32] EUROCAT Guide. 1.4 and reference documents. [cited 2017 October 11]. http://www.eurocat-network.eu/content/Full%20Guide%201%204%20version%2008_Sept2017.pdf.

[33] Galesloot TE, Vermeulen SH, Swinkels DW, de Vegt F, Franke B, den Heijer M, et al Cohort Profile: The Nijmegen Biomedical Study (NBS). Int J Epidemiol. 2017;46(4):1099–100j. Epub 2017/01/14. 10.1093/ije/dyw268 . 28082374 PMC5837647

[34] Exome Chip Design [cited 2018 05–03]. http://genome.sph.umich.edu/wiki/Exome_Chip_Design.

[35] Goldstein JI, Crenshaw A, Carey J, Grant GB, Maguire J, Fromer M, et al zCall: a rare variant caller for array-based genotyping: genetics and population analysis. Bioinformatics. 2012;28(19):2543–5. 10.1093/bioinformatics/bts479 . 22843986 PMC3463112

[36] Wu MC, Lee S, Cai T, Li Y, Boehnke M, Lin X. Rare-variant association testing for sequencing data with the sequence kernel association test. Am J Hum Genet. 2011;89(1):82–93. 10.1016/j.ajhg.2011.05.029 . 21737059 PMC3135811

[37] de Blaauw I, Wijers CH, Schmiedeke E, Holland-Cunz S, Gamba P, Marcelis CL, et al First results of a European multi-center registry of patients with anorectal malformations. J Pediatr Surg. 2013;48(12):2530–5. Epub 2013/12/10. 10.1016/j.jpedsurg.2013.07.022 . 24314198

[38] Grove ML, Yu B, Cochran BJ, Haritunians T, Bis JC, Taylor KD, et al Best practices and joint calling of the HumanExome BeadChip: the CHARGE Consortium. PLoS One. 2013;8(7):e68095 10.1371/journal.pone.0068095 . 23874508 PMC3709915

[39] Hong H, Xu L, Liu J, Jones WD, Su Z, Ning B, et al Technical reproducibility of genotyping SNP arrays used in genome-wide association studies. PLoS One. 2012;7(9):e44483 10.1371/journal.pone.0044483 . 22970228 PMC3436888

[40] Wong EH, Ng CL, Lui VC, So MT, Cherny SS, Sham PC, et al Gene network analysis of candidate loci for human anorectal malformations. PLoS One. 2013;8(8):e69142 10.1371/journal.pone.0069142 . 23936318 PMC3731316

[41] Saisawat P, Tasic V, Vega-Warner V, Kehinde EO, Gunther B, Airik R, et al Identification of two novel CAKUT-causing genes by massively parallel exon resequencing of candidate genes in patients with unilateral renal agenesis. Kidney Int. 2012;81(2):196–200. 10.1038/ki.2011.315 . 21900877 PMC3836012

[42] Svenningsson A, Gunnarsdottir A, Wester T. Maternal risk factors and perinatal characteristics of anorectal malformations. J Pediatr Surg. 2018;53(11):2183–8. Epub 2018/06/17. 10.1016/j.jpedsurg.2018.04.021 . 29907315

