# Supplementary material for: Exome chip association study excluded the involvement of rare coding variants with large effect sizes in the etiology of anorectal malformations
Source: PLoS One. 2019 May 28;14(5):e0217477. doi: 10.1371/journal.pone.0217477 (PMC6538182; doi:10.1371/journal.pone.0217477)
Supplement: S1 Fig — (PDF) [file pone.0217477.s001.pdf]

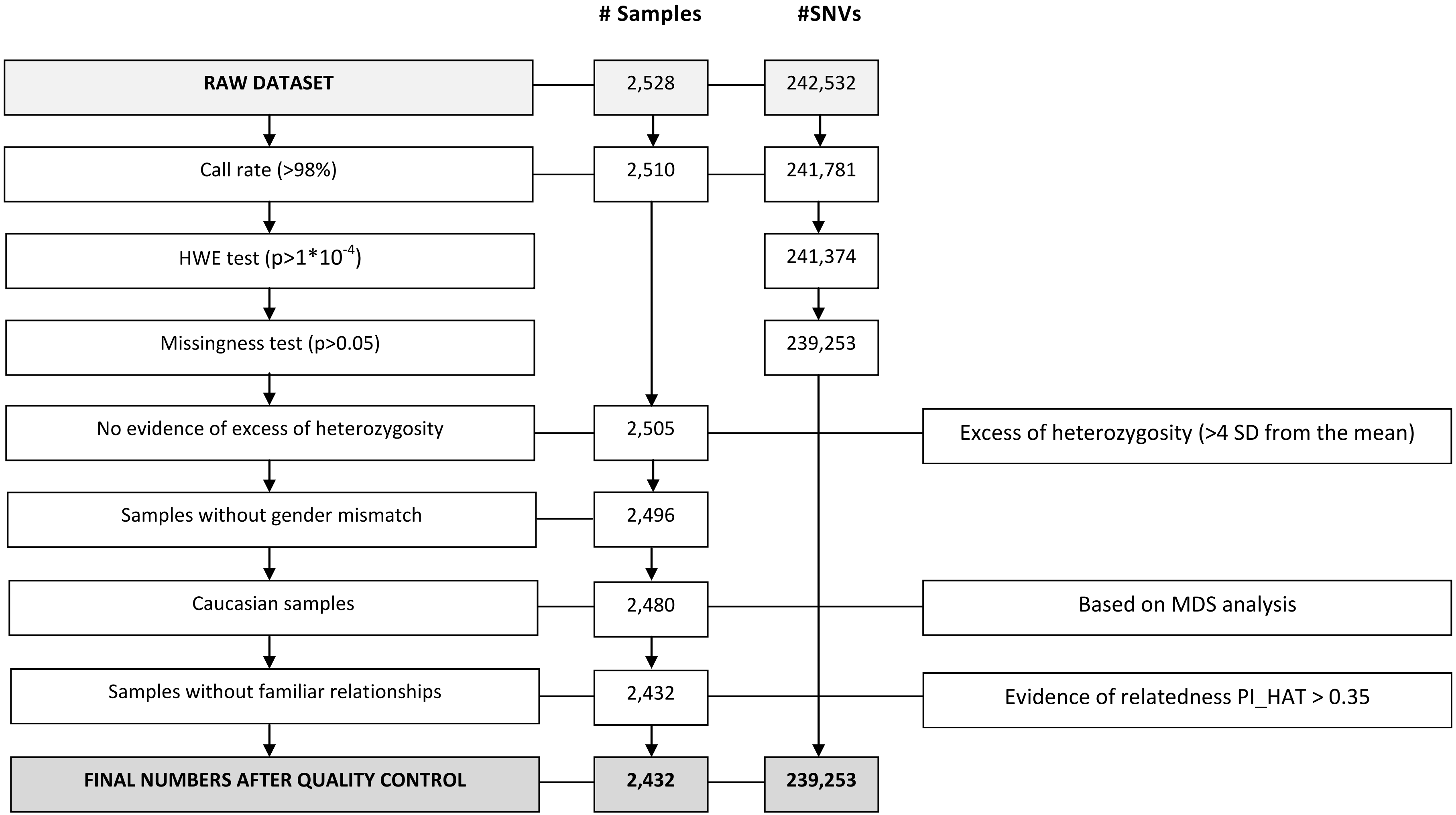


**S1 Figure. Summary of the quality control steps before the zCall procedure was applied.** SNV single nucleotide variant; HWE Hardy Weinberg Equilibrium; MDS multidimensional scaling analysis;
