# Supplementary material for: Exome chip association study excluded the involvement of rare coding variants with large effect sizes in the etiology of anorectal malformations
Source: PLoS One. 2019 May 28;14(5):e0217477. doi: 10.1371/journal.pone.0217477 (PMC6538182; doi:10.1371/journal.pone.0217477)
Supplement: S2 Fig — (PDF) [file pone.0217477.s002.pdf]

**S2 Figure. Manhattan plots.**

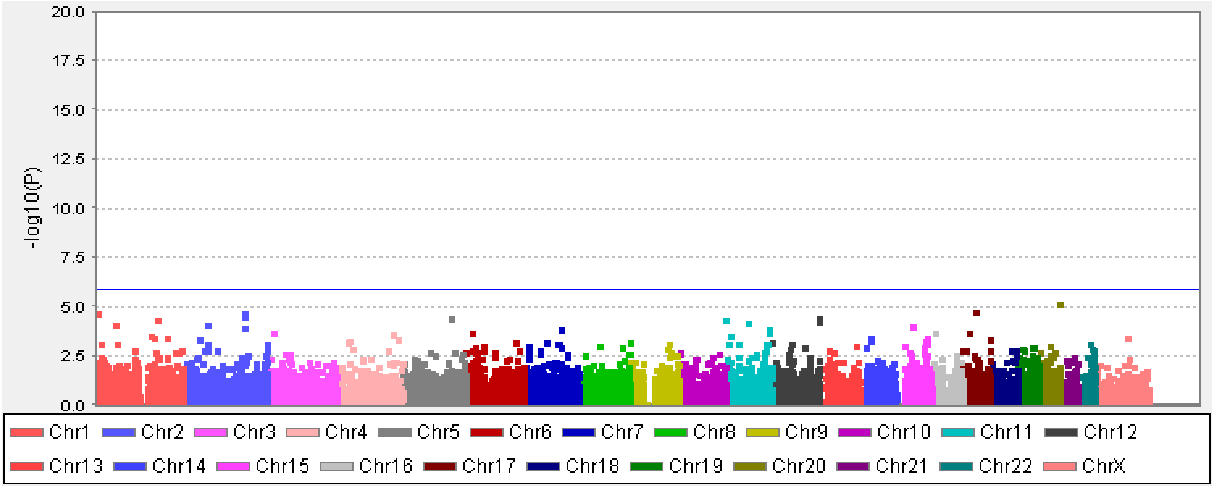


**Figure A.** Manhattan plot of the Fisher's exact test association p-values for the single variant analyses with MAF ≥1.0%. Chromosomes are shown on the x-axis. On the y-axis, the –log 10 of the *p*-value is shown. The blue line represents the threshold for significance after Bonferroni correction for 36,032 variants ($1.39*10^{-6}$, which corresponds to a value of 5.86). Variants above this line are considered statistically significant.

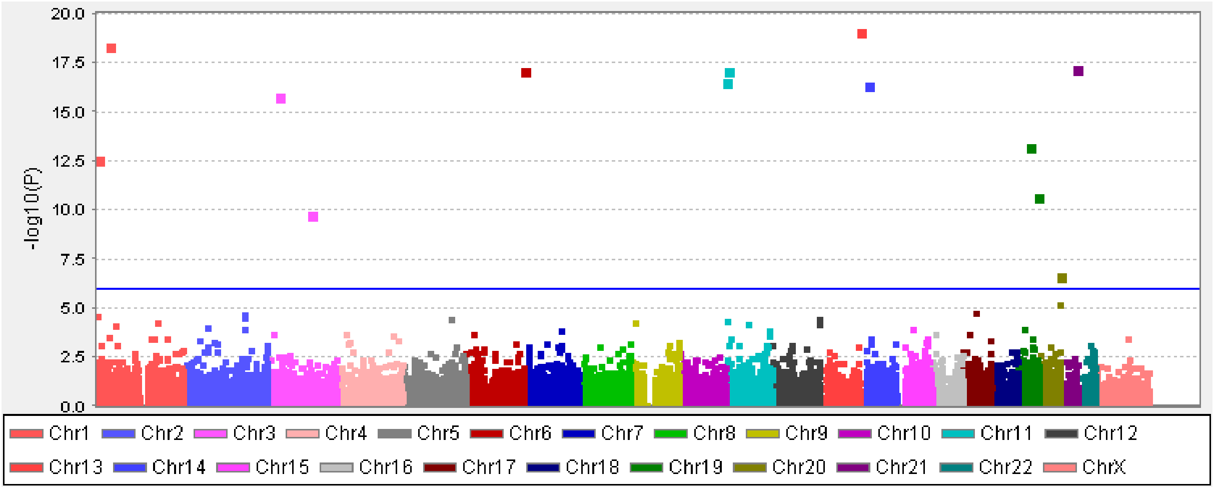


**Figure B.** Manhattan plot of the Fisher's exact test association p-values for the single variant analyses with MAF ≥0.4%. Chromosomes are shown on the x-axis. On the y-axis, the –log 10 of the *p*-value is shown. The blue line represents the threshold for significance after Bonferroni correction for 43,653 variants ($1.15*10^{-6}$, which corresponds to a value of 5.94). Variants above this line are considered statistically significant.
