# Supplementary material for: Exome chip association study excluded the involvement of rare coding variants with large effect sizes in the etiology of anorectal malformations
Source: PLoS One. 2019 May 28;14(5):e0217477. doi: 10.1371/journal.pone.0217477 (PMC6538182; doi:10.1371/journal.pone.0217477)
Supplement: S3 Fig — (PDF) [file pone.0217477.s003.pdf]

S3 Figure. Cluster plots.

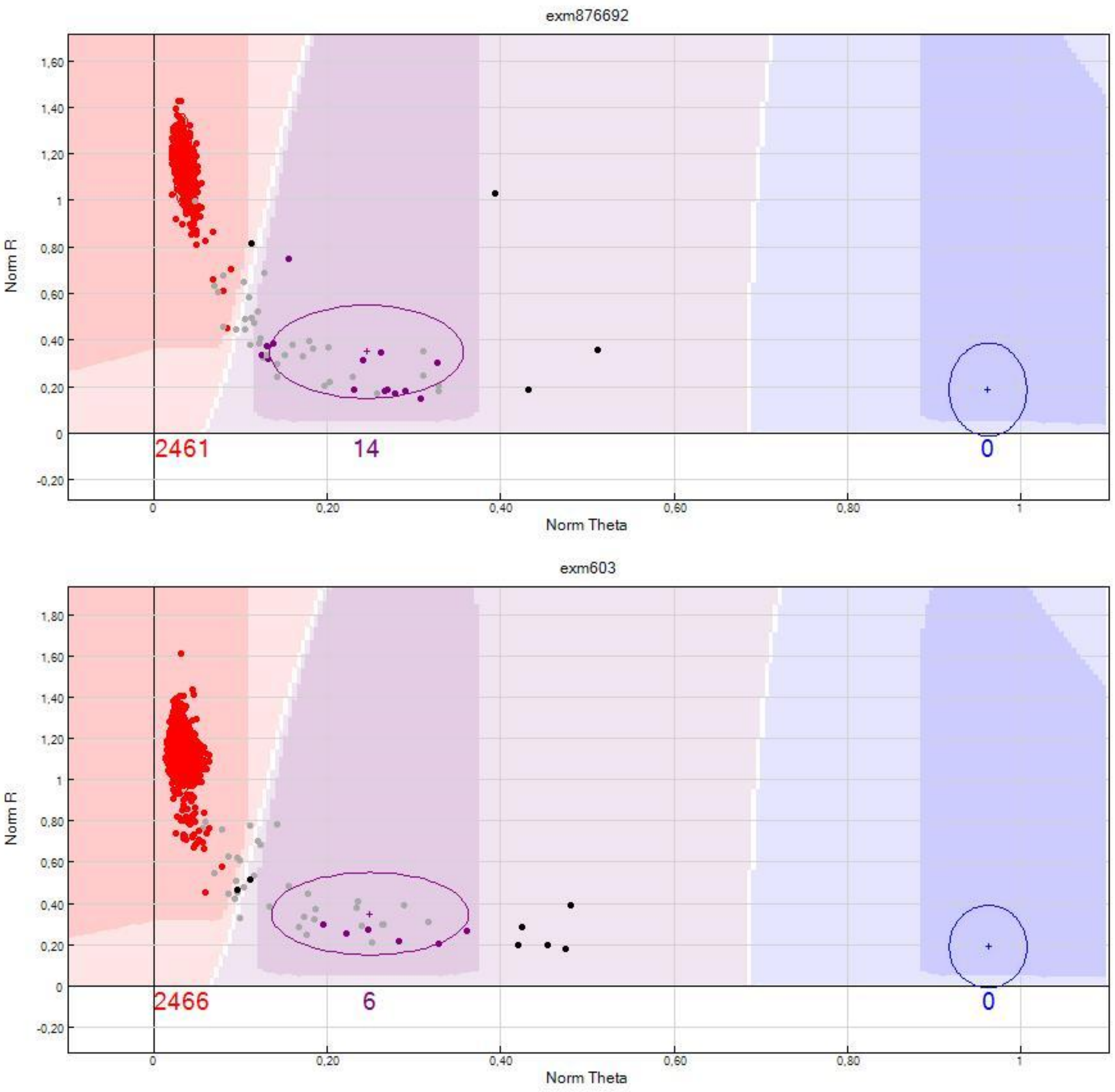

Figure A. Cluster plots of the variants with a mean normalized intensity (Norm R) below 0.4. The Y axis shows the signal intensity (Norm R) and the X-axis the allele frequency (Norm Theta). The homozygous wild type (shown in either blue or red) and indentified heterozygous group (shown in purple) are depicted within this cluster plot. The grey dots represent the samples with three or more risk alleles for the 13 statistically significant variants. The black dots represent samples that were not called by GenomeStudio.

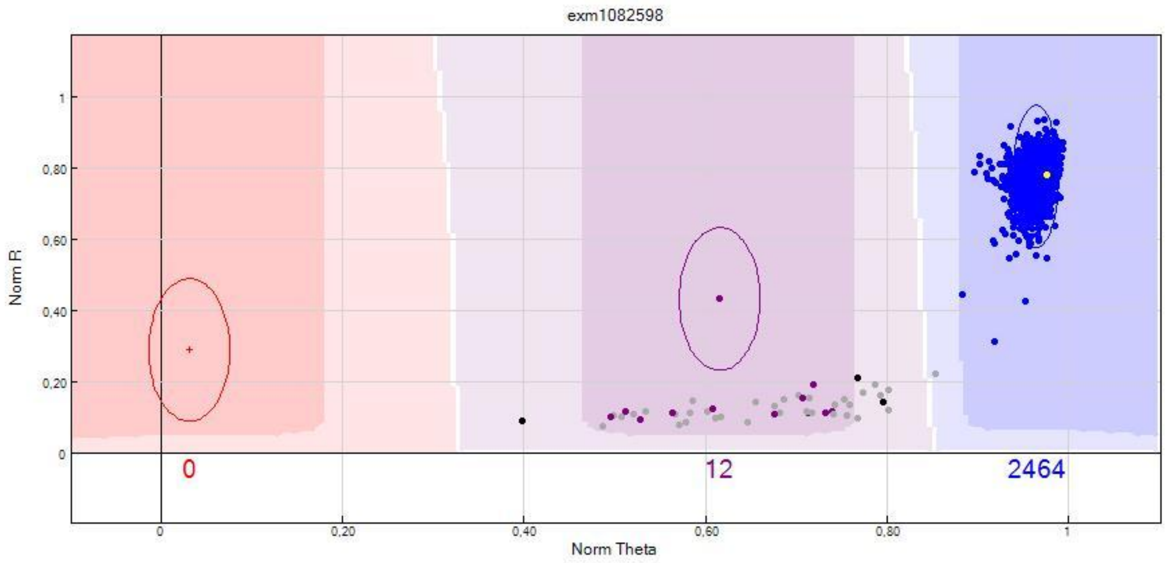
exm1082598
Norm R
Norm Theta
0
12
2464

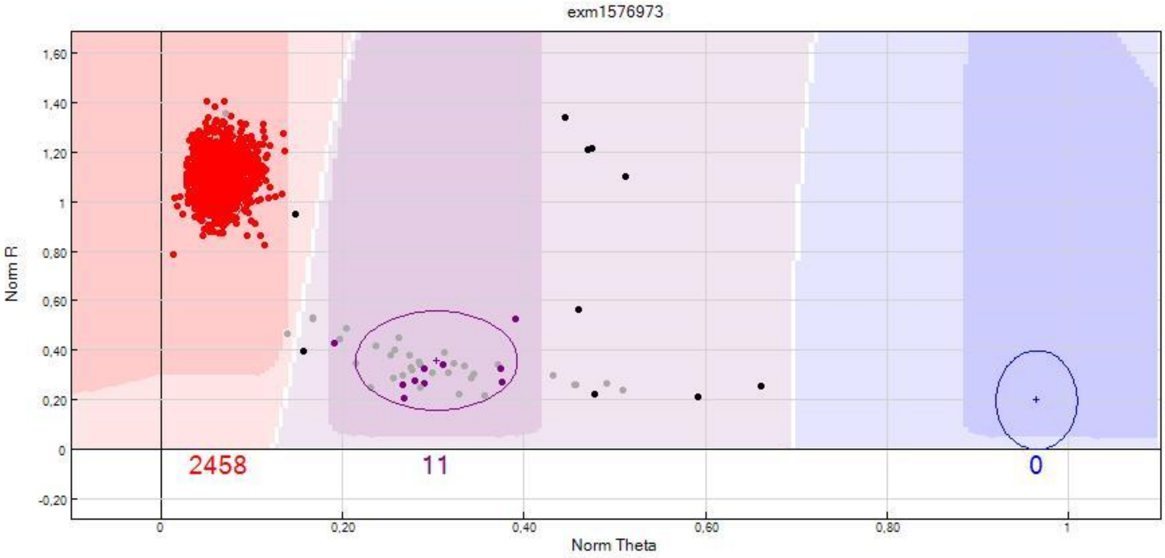
exm1576973
Norm R
Norm Theta
2458
11
0

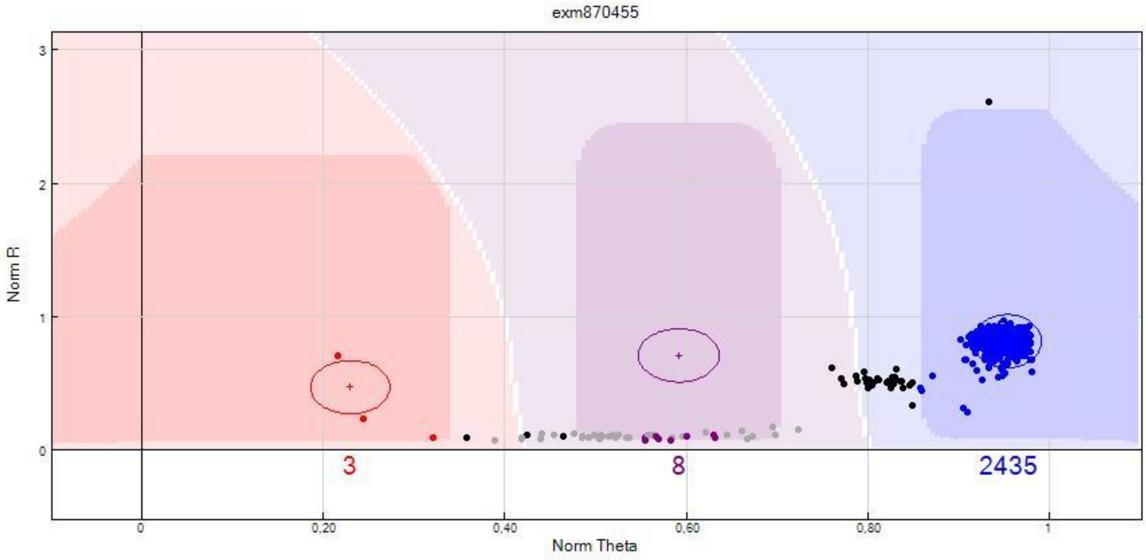
exm870455
Norm R
Norm Theta
3
8
2435

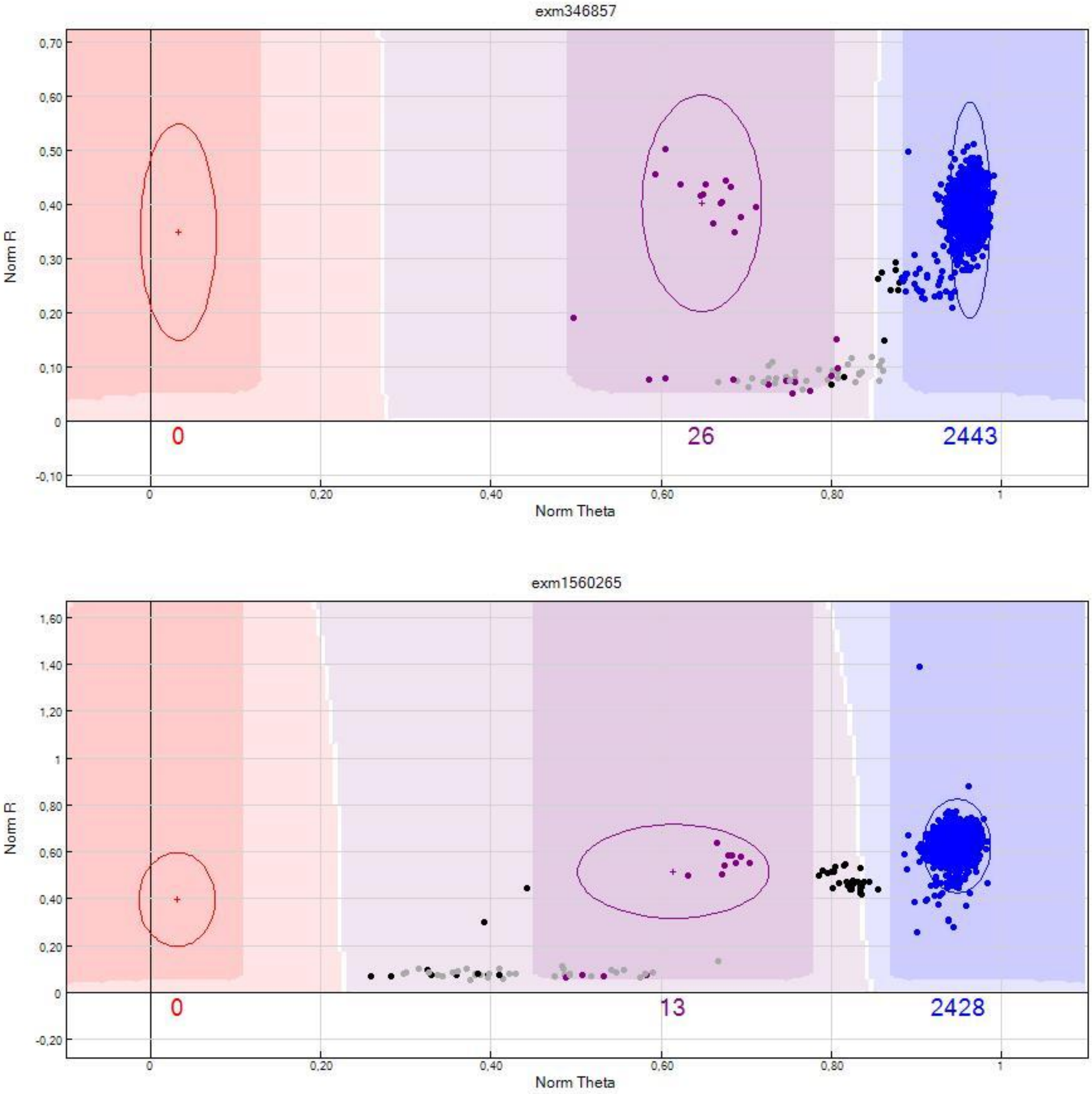

Figure B. Cluster plots of the variants with normalized intensity (Norm R) of individual samples below 0.4. The Y axis shows the signal intensity (Norm R) and the X-axis the allele frequency (Norm Theta). The homozygous wild type (shown in either blue or red) and indentified heterozygous group (shown in purple) are depicted within this cluster plot. The grey dots represent the samples with three or more risk alleles for the 13 statistically significant variants. The black dots represent samples that were not called by GenomeStudio.

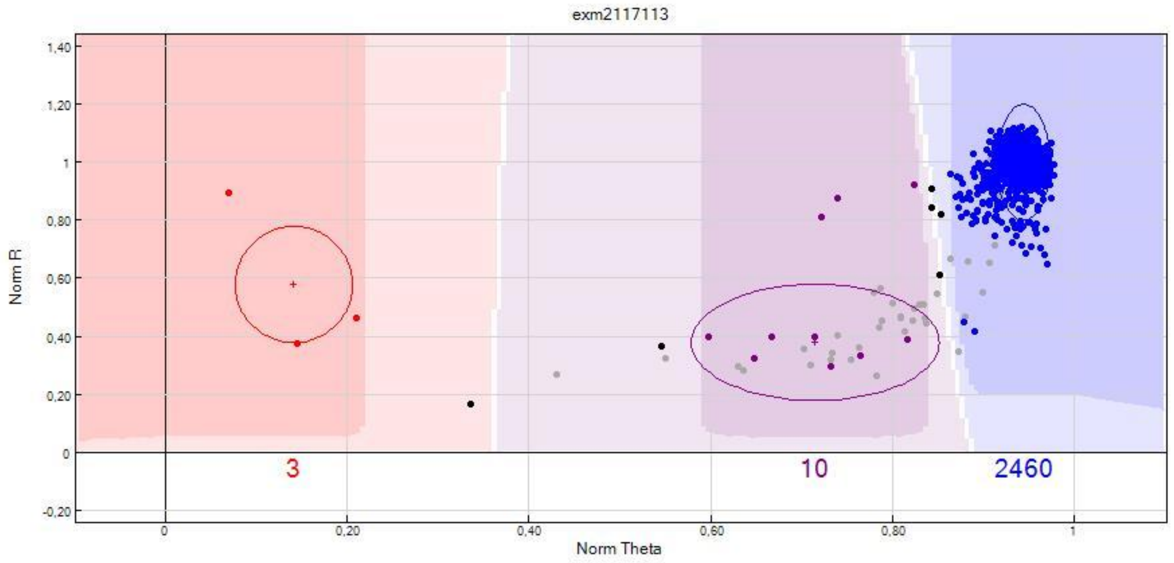
exm2117113
Norm R
Norm Theta
3
10
2460

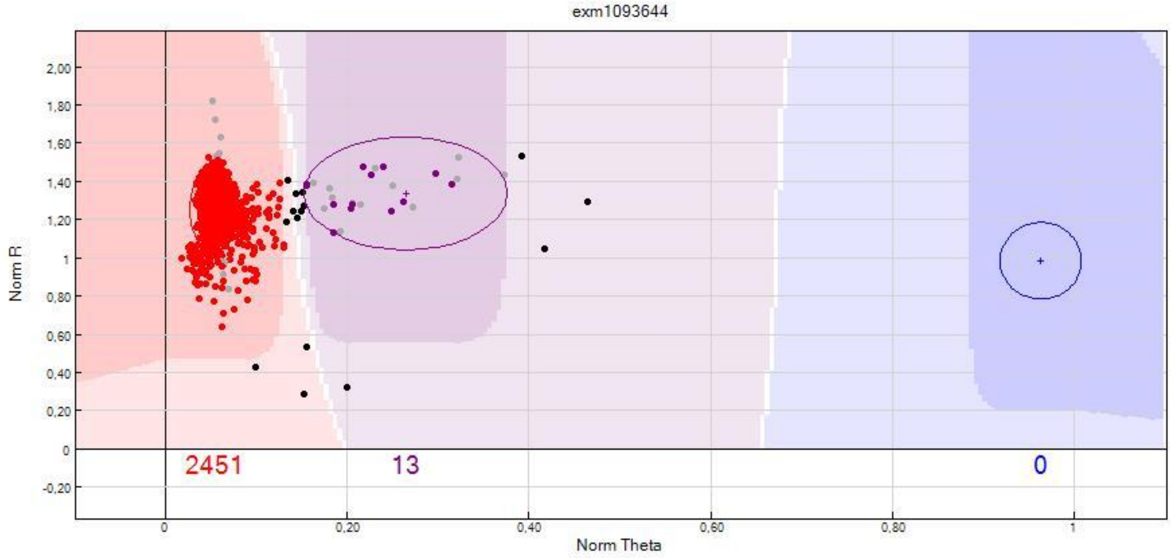
exm1093644
Norm R
Norm Theta
2451
13
0

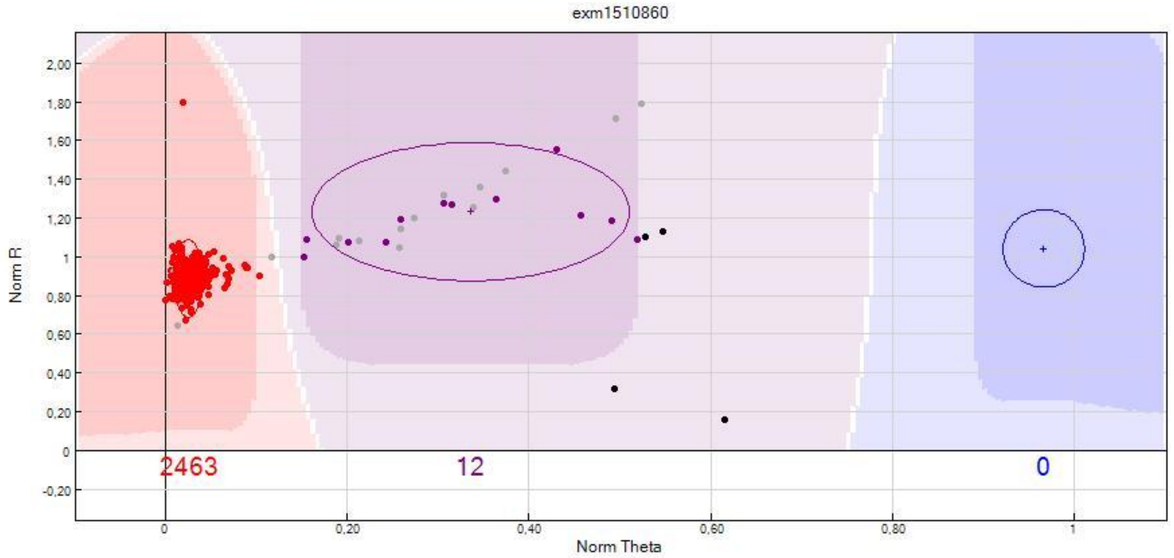
exm1510860
Norm R
Norm Theta
2463
12
0

Figure C. Cluster plots of the variants with a heterozygous genotype group consisting of ≥50% of multiple minor allele samples. The Y axis shows the signal intensity (Norm R) and the X-axis the allele frequency (Norm Theta). The homozygous wild type (shown in either blue or red) and indentified heterozygous group (shown in purple) are depicted within this cluster plot. The grey dots represent the samples with three or more risk alleles for the 13 statistically significant variants. The black dots represent samples that were not called by GenomeStudio.

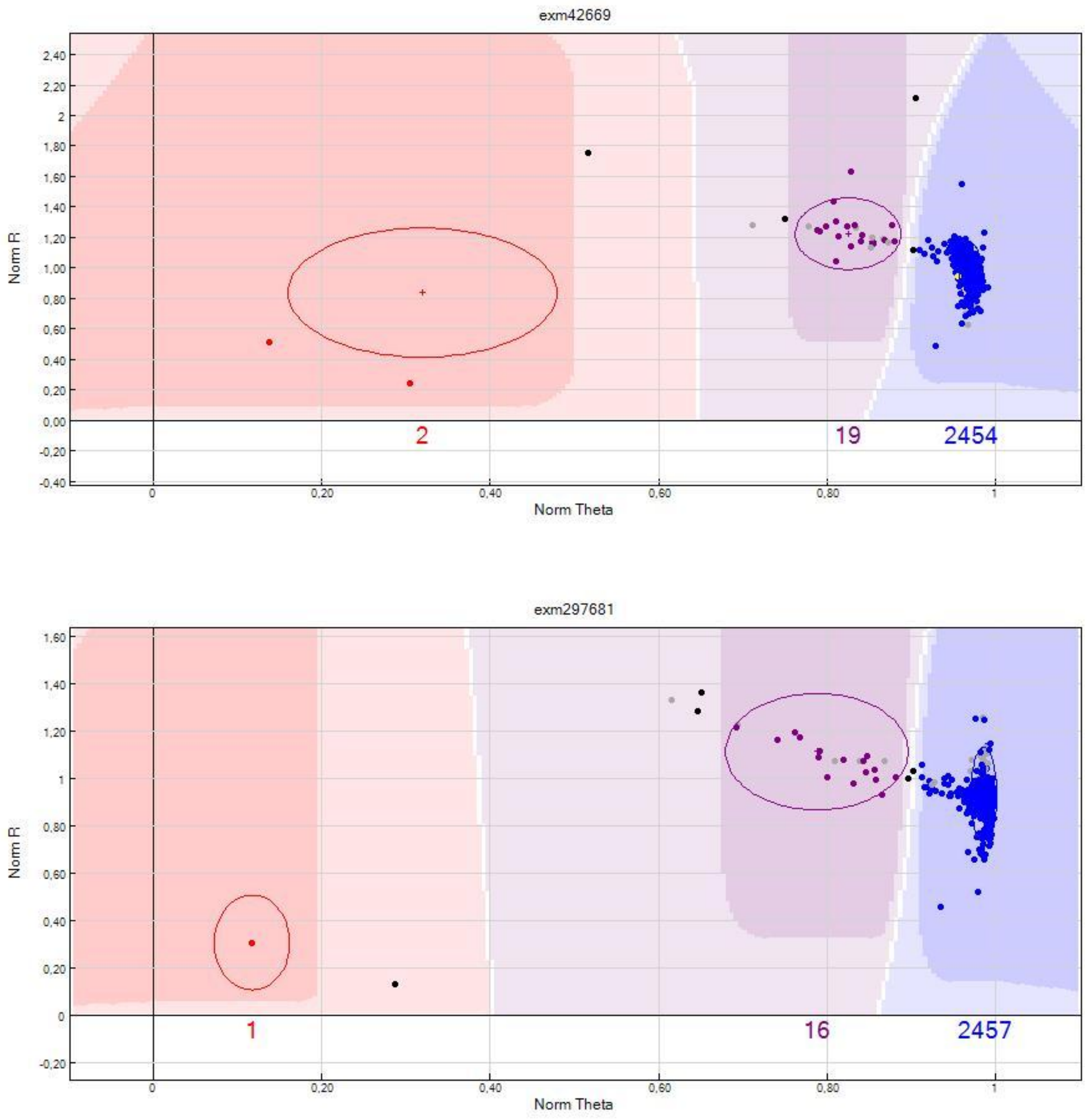

Figure D. Cluster plots of the two variants with acceptable calling quality. The Y axis shows the signal intensity (Norm R) and the X-axis the allele frequency (Norm Theta).The homozygous wild type (shown in blue) and indentified heterozygous group (shown in purple) are depicted within this cluster plot. Only a few homozygous variants were called for these two variants (shown in red). The grey samples represent the samples with three or more risk alleles for the 13 statistically significant variants. The black dots represent samples that were not called by GenomeStudio.

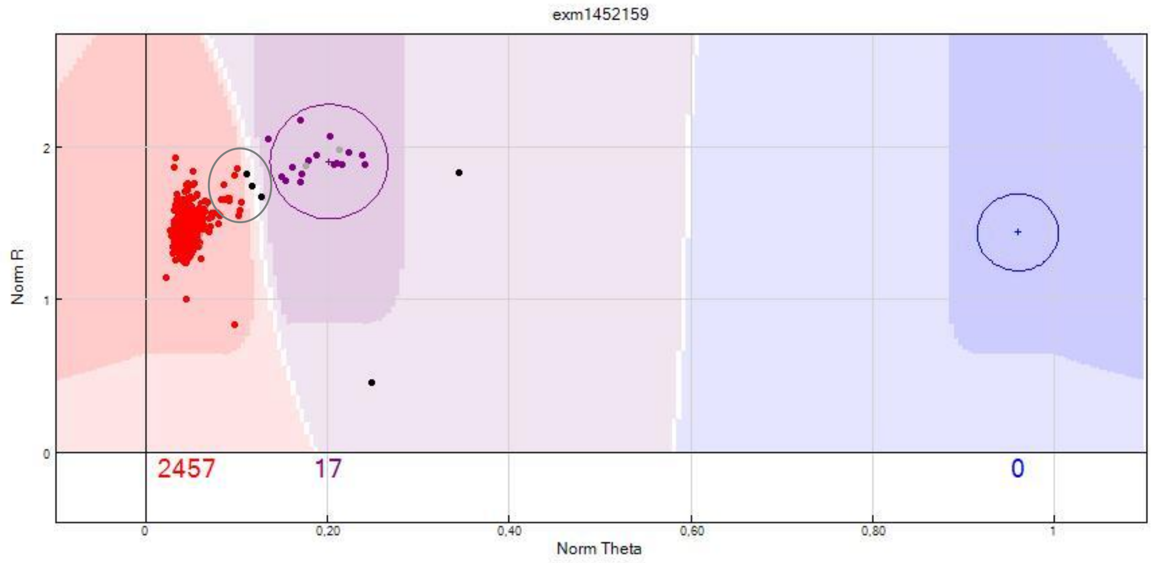

Figure E. Cluster plot of variant exm1452159. The Y axis shows the signal intensity (Norm R) and the X-axis the allele frequency (Norm Theta).The homozygous wild type (shown in red) and indentified heterozygous group (shown in purple) are depicted within this cluster plot. No homozygous variant was called for this variant. The grey samples represent the samples with three or more risk alleles for the 13 statistically significant variants. The black dots represent samples that were not called by GenomeStudio. Within the purple circle, the heterozygous genotypes originally called by GenomeStudio are shown. The grey circle shows the samples that potentially have the heterozygous genotype of this variant as well.
