# Supplementary material for: Exome chip association study excluded the involvement of rare coding variants with large effect sizes in the etiology of anorectal malformations
Source: PLoS One. 2019 May 28;14(5):e0217477. doi: 10.1371/journal.pone.0217477 (PMC6538182; doi:10.1371/journal.pone.0217477)
Supplement: S1 Table — (DOCX) [file pone.0217477.s004.docx]

**S1 Table. Statistically significant (but false positive) associations between anorectal malformations and single variants (MAF value ≥ 0.4%), genotypic model.**

| **ID** | **Chr** | **Position^a^** | **rs-id** | **Minor / Major allele** | **Protein change** | **Gene** | **Genotype Controls**  **(AA/AB/BB)**^b^ | **Genotype**  **Patients**  **(AA/AB/BB)^b^** | ***P*^c^** |
| --- | --- | --- | --- | --- | --- | --- | --- | --- | --- |
| exm1082598 | 13 | 114537621 | rs78824256 | T/C | Arg246His | *GAS6^d^, GAS6-AS1* | 0/30/533 | 0/0/1860 | 1.04x10^-18^ |
| exm42669 | 1 | 34330070 | rs144223004 | T/C | Pro93Leu | *HMGB4^d^, CSMD2* | 0/29/539 | 0/0/1860 | 5.08x10^-18^ |
| exm1576973 | 21 | 45821582 | rs9974927 | G/T | Asp780Glu | *TRPM2* | 0/31/533 | 0/2/1858 | 5.86x10^-17^ |
| exm2117113 | 6 | 169642042 | rs202062355 | T/C | Ala236Thr | *THBS2* | 0/27/540 | 0/0/1860 | 7.93x10^-17^ |
| exm876692 | 11 | 1281885 | rs113740363 | G/A | Ile5666Val | *MUC5B* | 0/27/541 | 0/0/1860 | 8.21x10^-17^ |
| exm870455 | 11 | 403981 | rs202227463 | A/G | Gly721Ser | *PKP3* | 0/28/531 | 0/1/1859 | 2.71x10^-16^ |
| exm1093644 | 14 | 24780216 | rs201778907 | G/A | Ser116Gly | *CIDEB, LTB4R2^d^* | 0/28/540 | 0/1/1859 | 3.76x10^-16^ |
| exm297681 | 3 | 33195264 | rs75287757 | T/C | Arg287Gln | *SUSD5* | 0/25/543 | 0/0/1860 | 1.32x10^-15^ |
| exm1452159 | 19 | 32844890 | rs150068736 | G/T | Ile385Ser | *ZNF507* | 0/21/547 | 0/0/1860 | 3.35x10^-13^ |
| exm603 | 1 | 900519 | rs140019196 | G/A | Asn626Ser | *KLHL17* | 0/20/548 | 0/0/1860 | 1.36x10^-12^ |
| exm1510860 | 19 | 56487619 | rs61734100 | G/C | Ile942Met | *NLRP8* | 0/23/545 | 0/4/1856 | 7.69x10^-11^ |
| exm346857 | 3 | 126236523 | rs147828466 | A/G | Arg14Trp | *UROC1* | 0/26/528 | 0/9/1851 | 5.73x10^-10^ |
| exm1560265 | 20 | 62324609 | rs139221232 | T/C | Arg989Trp | *RTEL1* | 0/18/529 | 0/7/1853 | 5.65x10^-7^ |

Chr: chromosome; MAF: minor allele frequency; ^a^ Genome positions are based on human genome build hg19; ^b^AA = homozygous reference genotype, AB = heterozygous genotype, BB = homozygous variant genotype; ^c^ *P* value calculated for a **genotypic model** using Fisher’s exact test and adjusted for genomic control (lambda=1.072); ^d^ Variant affects the coding region of this gene, but intronic region in other gene.
