# Supplementary material for: Exome chip association study excluded the involvement of rare coding variants with large effect sizes in the etiology of anorectal malformations
Source: PLoS One. 2019 May 28;14(5):e0217477. doi: 10.1371/journal.pone.0217477 (PMC6538182; doi:10.1371/journal.pone.0217477)
Supplement: S2 Table — (DOCX) [file pone.0217477.s005.docx]

**S2 Table**. **Associations between anorectal malformations and genes with at least two variants with a MAF ≥1.0%.**

| **Gene** | **Number of variants**  **on exome chip** | ***p*-value based**  **on SKAT analyses** |
| --- | --- | --- |
| *DUT* | 2 | 2.15 x 10^-05^ |
| *PCDH1* | 4 | 9.04 x 10^-05^ |
| *HDGF* | 2 | 1.00 x 10^-04^ |
| *WDR64* | 7 | 1.21 x 10^-04^ |
| *TNN* | 4 | 1.22 x 10^-04^ |
| *ACTN3* | 8 | 2.82 x 10^-04^ |
| *SCRIB.SCRIB* | 6 | 5.10 x 10^-04^ |
| *T ENC1* | 2 | 6.13 x 10^-04^ |
| *MPHOSPH10* | 3 | 6.25 x 10^-04^ |
| *BTD* | 4 | 7.09 x 10^-04^ |
| *KIAA0556* | 6 | 7.69 x 10^-04^ |
| *LAMA5* | 10 | 8.85 x 10^-04^ |
| *TFAP2B* | 2 | 9.41 x 10^-04^ |
| *ERV3-1* | 7 | 1.03 x 10^-03^ |
| *USP36* | 5 | 1.20 x 10^-03^ |
| *NR3C2* | 4 | 1.33 x 10^-03^ |
| *IP6K2* | 2 | 1.37 x 10^-03^ |
| *NAALAD2* | 7 | 1.46 X 10^-03^ |
| *ATP1B2* | 2 | 1.53 x 10^-03^ |
| *TOP1* | 3 | 2.02 x 10^-03^ |
| *IRF4* | 2 | 2.24 x 10^-03^ |
| *LCP2* | 2 | 2.25 x 10^-03^ |
| *GLT8D2* | 2 | 2.27 x 10^-03^ |
| *OR6N1* | 6 | 2.33 x 10^-03^ |
| *ZNF616* | 4 | 2.34 x 10^-03^ |
| *ATP13A2* | 3 | 2.44 x 10^-03^ |
| *C1orf168* | 3 | 2.44 x 10^-03^ |
| *ZNF516* | 5 | 2.54 x 10^-03^ |
| *ADAM22* | 3 | 2.81 x 10^-03^ |
| *HDAC7* | 2 | 2.86 x 10^-03^ |
| *MATN4* | 2 | 2.93 x 10^-03^ |
| *EDEM2* | 3 | 2.98 x 10^-03^ |
| *AVPI1* | 3 | 2.98 x 10^-03^ |
| *FCN2* | 2 | 3.04 x 10^-03^ |
| *PFKP* | 2 | 3.33 x 10^-03^ |
| *TAS1R3* | 3 | 3.39 x 10^-03^ |
| *OR5V1* | 3 | 3.39 x 10^-03^ |
| *AR* | 2 | 3.40 x 10^-03^ |
| *WDR90* | 8 | 3.49 x 10^-03^ |
| *C22orf40* | 3 | 3.50 x 10^-03^ |
| *ADAMTS9-AS2.MIR548A2* | 2 | 3.62 x 10^-03^ |
| *DCAKD* | 2 | 3.67 x 10^-03^ |
| *ACVR1C* | 4 | 3.87 x 10^-03^ |
| *GPT* | 2 | 3.92 x 10^-03^ |
| *ANPEP* | 4 | 3.94 x 10^-03^ |
| *BTN3A2* | 2 | 4.18 x 10^-03^ |
| *GRM1.GRM1* | 5 | 4.19 x 10^-03^ |
| *SYT9* | 4 | 4.68 x 10^-03^ |
| *CTIF.CTIF* | 2 | 4.68 x 10^-03^ |
| *ADAMTS9-AS2* | 2 | 4.89 x 10^-03^ |
| *C15orf2* | 6 | 4.91 x 10^-03^ |
| *GRHL1* | 3 | 5.53 x 10^-03^ |
| *MEIS2* | 4 | 6.06 x 10^-03^ |
| *PARP2.PARP2* | 3 | 6.08 x 10^-03^ |
| *LOC100128496.C20orf79* | 2 | 6.10 x 10^-03^ |
| *SLC7A9* | 3 | 6.21 x 10^-03^ |
| *SLC26A8* | 6 | 6.39 x 10^-03^ |
| *GPSM2* | 3 | 6.43 x 10^-03^ |
| *ZNF33A.ZNF33A* | 2 | 6.45 x 10^-03^ |
| *C19orf55* | 3 | 6.47 x 10^-03^ |
| *SERINC2* | 2 | 6.86 x 10^-03^ |
| *KANK1* | 6 | 6.91 x 10^-03^ |
| *EXD3* | 3 | 6.92 x 10^-03^ |
| *C9orf68* | 4 | 7.17 x 10^-03^ |
| *TEKT3* | 2 | 7.36 x 10^-03^ |
| *SEMA4G.MRPL43* | 3 | 7.59 x 10^-03^ |
| *ST3GAL6* | 2 | 7.65 x 10^-03^ |
| *TNS3* | 6 | 7.76 x 10^-03^ |
| *ARMC5* | 2 | 7.89 x 10^-03^ |
| *PITRM1* | 9 | 7.97 x 10^-03^ |
| *KALRN* | 7 | 7.97 x 10^-03^ |
| *ZNF837* | 2 | 8.14 x 10^-03^ |
| *ZNF44* | 2 | 8.17 x 10^-03^ |
| *EGFEM1P* | 3 | 8.62 x 10^-03^ |
| *FLJ43860* | 21 | 8.75 x 10^-03^ |
| *FAM123C* | 2 | 8.91 x 10^-03^ |
| *ZNF134* | 2 | 8.91 x 10^-03^ |
| *STX17* | 2 | 9.08 x 10^-03^ |
| *TMPRSS15* | 4 | 9.37 x 10^-03^ |
| *TM6SF1* | 2 | 9.56 x 10^-03^ |
| *FAM3B* | 3 | 9.93 x 10^-03^ |

Genes in this table have a *p*-value < 0.01. None of the gene findings remained statistically significant after Bonferroni correction for the number of genes tested (N=6,188; *p* < 8.08 x 10^-06^).
