# Supplementary material for: Exome chip association study excluded the involvement of rare coding variants with large effect sizes in the etiology of anorectal malformations
Source: PLoS One. 2019 May 28;14(5):e0217477. doi: 10.1371/journal.pone.0217477 (PMC6538182; doi:10.1371/journal.pone.0217477)
Supplement: S1 Text — (DOCX) [file pone.0217477.s006.docx]

**S1 Text. Additional information on methods and results.**

**Methods**

**Extra quality control step**

We studied the 13 variants that were statistically significant in the single variant analyses (MAF ≥0.4%) in further detail by visualizing the corresponding cluster plots in GenomeStudio (version 2.0). Based on visual inspection of the signal intensities (Norm R), we re-evaluated the calling quality. Variants with a mean Norm R below 0.2 were already removed during the calling process in GenomeStudio. However, variants with a mean Norm R below 0.4 may also be considered technical errors, according to the Illumina Genotyping Data Analysis Guide(1). Therefore, we applied these more stringent criteria and excluded the variants with a mean Norm R below 0.4 (Figure A S3 Fig).

In addition, we checked the Norm R of the individual samples in the clusterplots, as the mean Norm R value for a specific cluster (indicated in the middle of the coloured circles with a ‘+’) can be clearly different from the location of the individual samples that belong to that cluster (Figure B S3 Fig). Variants for which the majority of the individual samples had a Norm R below 0.4 were excluded as candidates for validation.

We also observed that some samples seemed to contain 3 or more minor alleles of the 13 rare statistically significant variants (MAF value ≥0.4%) (subsequently referred to as: the multiple minor allele samples). We assumed that having 3 or more of these minor alleles would be very unlikely, based on the population frequencies of these variants in the ExAC database (Table 2). Most likely, the calling quality for these samples was poor, perhaps due to poor DNA quality. We depicted the multiple minor allele samples within the cluster plots by grey dots. Based on an arbitrary cut-off, we considered variants with a heterozygous genotype cluster consisting of ≥50% of these multiple minor allele samples as not being reliably called and therefore also as technical errors.

**Sanger sequencing**

Forward and reverse primers were designed using the UCSC genome browser (Hg38) and Primer3Plus(2) to sequence the variant itself and the surrounding 100 base pairs. Polymerase chain reaction (PCR) was carried out in a 7.5 µl reaction volume containing 0.25 µl of the forward and reverse primer (10 pmol/µl), 1.0 µl genomic DNA (10 ng/µl), 2.25 µl Milli-Q, and 3.75 µl AmpliTaq Gold® 360 PCR Master Mix (Applied Biosystems). The PCR conditions were 95°C for 10 min followed by 35 cycles of: 95°C for 30 s, 56°C (annealing temperature) for 30 s, and 72°C for 1 min, followed by a final extension step of 72°C for 7 min. The PCR was performed using a Dyad Peltier Thermal Cycler (BioRad). Using ExoSAP-IT(3), the PCR products were purified and subsequently, 1 µl of purified PCR product was unidirectional sequenced using Sanger sequencing on a 3730xl DNA sequencer (Applied Biosystems). Experiments were performed at the Radboudumc, Nijmegen, the Netherlands, in a laboratory recognized and granted accreditation for quality control by the coordinating committee for improvement of quality control of laboratory research in health care.

**Results**

**Extra quality control step**

The mean Norm R of the heterozygous cluster was <0.4 for two variants (exm876692 and exm603), so these variants were excluded as candidates for validation (Figure A S3 Fig). Five other variants had a mean Norm R above 0.4, but the majority of the individual samples that were part of the heterozygous cluster were clearly below that Norm R value and were therefore also excluded as candidates for validation (exm1082598, exm1576973, exm870455, exm346857, and exm1560265) (Figure B S3 Fig).

In total, 53 patient samples and 0 control samples were identified as multiple minor allele samples. The visualization of the multiple minor allele samples in the cluster plots identified three additional variants as technical errors (exm2117113, exm1093644, exm1510860), as the samples with multiple minor alleles were very prevalent within the heterozygous genotype cluster (≥50%), (Figure C S3 Fig). We also excluded these three variants, and therefore we excluded 10 out of the 13 statistically significant variants as candidates for validation based on technical errors.

Two of the three remaining variants showed acceptable calling quality based on the intensity of calling, and had less than 50% of multiple minor allele samples within their heterozygous genotype cluster (Figure D S3 Fig). The first variant, exm42269, corresponding to SNV rs144223004 in the gene High Mobility Group Box 4 (*HMGB4)* reached a *p-*value of 5.71x10^-18^ in the single variant analyses. The heterozygous genotype for this variant was observed in 29 patients and none of the controls. For the second variant, exm297681, corresponding to SNV rs75287757 in the gene Sushi Domain Containing 5 (*SUSD5)* a p-value of 1.41x10^-15^ was observed for the association with ARM. The heterozygous genotype for this variant was observed in 24 patients and none of the controls.

For the third remaining variant, we hypothesized that the cluster of samples on the left side of the heterozygous genotype cluster (Figure E S3 Fig) might also have the heterozygous genotype instead of the homozygous wild type genotype, since those samples are somewhat separately located in between both clusters. This variant, exm1452159, corresponds to SNV rs150068736 in the gene Zinc Finer Protein 507 (*ZNF507).* In the single variant analysis, a *p-*value of 3.44x10^-13^ was observed for the association of this variant with ARM. The heterozygous genotype of this variant was observed in 21 patients and none of the controls. When we included the samples of the additional cluster on the left side of the heterozygous genotype cluster, we had 31 patients and no controls for validation of this variant.

**References**

1. Illumina. *Infinium Genotyping Data Analysis - A guide for analyzing Infinium genotyping data usingGenomeStudio Genotyping Module*. Available from: https://www.illumina.com/Documents/products/technotes/technote_infinium_genotyping_data_analysis.pdf.

2. Untergasser, A., et al., *Primer3Plus, an enhanced web interface to Primer3.* Nucleic Acids Res,2007. **35**(Web Server issue): p. W71-4.

3. Bell, J., *A simple way to treat PCR products prior to sequencing using ExoSAP-IT.* Biotechniques, 2008. **44**(6): p. 834.
